# Outcome measurement in functional neurological disorder: A qualitative study on the views of patients, caregivers and healthcare professionals

**DOI:** 10.1007/s00415-025-12912-9

**Published:** 2025-02-11

**Authors:** Sonja Rutten, Abigail Bradley-Westguard, Timothy R. Nicholson, Susannah Pick

**Affiliations:** 1 Department of Psychiatry, Amsterdam University Medical Center, Amsterdam, the Netherlands; 2 Institute of Psychiatry, Psychology & Neuroscience, King’s College London, London, UK

**Keywords:** Conversion disorder, Outcome assessment, Qualitative research

## Abstract

**Background:**

In this qualitative study, we aimed to obtain and synthesise the views of patients with functional neurological disorder (FND), their caregivers, and relevant healthcare professionals (HCPs) on outcome measurement in FND.

**Methods:**

Semi-structured interviews were conducted with 22 FND patients, 18 caregivers and 21 HCPs, sampled purposively in the United Kingdom. Transcripts were analysed through inductive thematic analysis.

**Results:**

Whilst reduction or resolution of FND symptoms were frequently mentioned as important treatment goals in all groups, this was reported by a larger proportion of caregivers and HCPs than patients. Patients most frequently hoped for improvements in mental health/well-being. Other important treatment goals were resuming work, and an increase in independence, self-management or self-efficacy.

Of the 20 domains deemed relevant for outcome assessment, improvements in FND symptoms, emotional well-being, activities of daily living and quality-of-life, were mentioned most frequently.

None of the participants thought that outcome assessment should be purely clinician-rated or objective; all believed that the patient’s subjective experience should be central. Nevertheless, participants in all groups acknowledged that clinician-rated or objective OMIs have added value in clinical outcome assessment. The benefits of digital outcome assessment were also mentioned by several participants.

**Conclusions:**

This is the first study to capture the views of key stakeholders on outcome assessment in FND. The findings indicate that outcome measures for FND should be patient-centred, whilst also including HCP opinion. Critical domains for assessment are FND symptoms, mental health, quality-of-life and the ability to perform activities of daily living.

**Supplementary Information:**

The online version contains supplementary material available at 10.1007/s00415-025-12912-9.

## Background

Functional neurological disorder (FND) is defined by neurological symptoms with clinical features that are incongruent with structural neurological abnormalities, causing significant distress and functional impairment [1]. FND is diagnosed based on ‘core’ neurological symptoms, but most patients also experience associated physical and psychological symptoms, including pain, fatigue, depression and anxiety [2]. FND can affect multiple life domains and results in a decreased quality-of-life [3, 4].

Although scientific and clinical interest in FND is rapidly increasing, there are few evidence-based treatments. A key component of evidence-based medicine, especially treatment trials, is outcome assessment with valid and reliable outcome measurement instruments (OMIs). However, there has been a paucity of research aimed at the development of OMIs for FND. As a result, studies have used a wide variety of different OMIs in FND trials [5]. In 2017, international FND experts formed the FND Core Outcome Measure (FND-COM) group [5, 6] to work towards consensus recommendations for outcome measurement in FND. They evaluated the OMIs used previously in FND clinical trials, concluding that all existing FND-specific OMIs showed substantial clinimetric limitations [5]. They also acknowledged the importance of measuring the impact of FND on other domains, including daily functioning, quality-of-life and psychological well-being. In the absence of widely endorsed OMIs for FND research, they produced an interim consensus core outcome set [5]. However, involvement of patients and their caregivers in this process was restricted to representation of the major FND charitable organisations in the FND-COM group.

Although there is increasing recognition that patient and caregiver views on outcome measures are a key part of the process of developing a core outcome set and OMIs [7, 8] there have been no previous studies of patient or caregiver perspectives on outcome measurement for FND. We therefore performed a qualitative study to obtain more insight into the experiences of people with FND, their caregivers, and health care professionals (HCPs) working with FND, specifically in relation to measurement of outcomes and recovery. Here, we report the results relevant to the development of an FND-specific OMI and core outcome set, including: treatment goals, outcomes deemed relevant for FND clinical assessment and views on how, and by whom, these should be assessed.

## Methods

### Design

In this qualitative study, semi-structured interviews were conducted with FND patients, caregivers and HCPs. This study was approved by the West Midlands-Solihul National Health Service Research Ethics Committee, UK (reference number 18/WM/0110).

### Participants

Between 20 and 25 participants were recruited from the following groups: (i) FND patients, (ii) caregivers of FND patients, i.e. individuals who provide support/care for an FND patient in daily life and (iii) HCPs with experience of working directly with FND patients. Participants were selected purposively including FND patients with various symptom subtypes and disease duration, caregivers with different types of relationships to the patient (partners, relatives), and HCPs with diverse professional backgrounds and levels of experience.

The inclusion criteria for participation were:

- Aged 18 years or above.
- Fluent in English.
- Willing and able to give informed consent.
- For patients: diagnosed with FND by a neurologist/neuropsychiatrist.
- For caregivers: providing support or care for an FND patient in daily life.
- For HCPs: at least one year of experience working directly with FND patients.

FND patients were excluded if they had any current major psychiatric (i.e. psychosis, substance dependence) or neurological (e.g. epilepsy, neurodegenerative disease) disorder, to ensure that the information elicited was specific to living with FND.

### Study procedures

Recruitment of FND patients and caregivers took place at clinics within neuropsychiatry services and through patient support organisations in the UK. They were either approached by a member of the clinical team or contacted the lead researcher directly (SP) in response to an advert. HCPs were recruited with a flyer shared within professional networks in the UK, using email circular lists and via social media and websites from patient support organisations. Potential participants who approached the researcher in response to the advert received a brief overview of the project and a Participant Information Sheet. All participants were given the opportunity to ask questions before deciding to participate. Written informed consent was obtained by means of participant dated signature.

Eligibility of patients was confirmed by SP based on medical records/correspondence, provided by the patient. Caregivers and HCPs were screened for eligibility by SP during a screening interview by telephone or in clinic. SP did not have a clinical or professional relationship with the study participants prior to the study.

Interviews were conducted once per participant, lasting between 30 and 90 min. Participants were interviewed individually at the location where they felt most comfortable, i.e. at their own home, workplace, or an interview room at King’s College London. All interviews were conducted by SP following interview guides, developed in consultation with FND patient representatives and the FND-COM group. Interviews were audio recorded and transcribed verbatim by SP.

### Data analysis

This study aimed to accurately reflect the subjective experiences of FND patients, their caregivers and HCPs, rather than to develop a novel theoretical perspective from the interpretations of the analyst. Therefore, inductive thematic analysis was used for data analysis, adopting a pragmatic, data-driven, realist/essentialist perspective [9].

Three data coders independently coded a subset of transcripts: SP (academic psychologist, senior research fellow), ABW (trained patient research collaborator) and SR (psychiatrist, senior researcher). All data coders had previous experience with qualitative analyses. Multiple coding was ensured by the analysis of a subset of at least five transcripts by the other data coders.

Each data coder first familiarised herself with the transcripts by reading them several times. Next, the transcripts were separated into meaningful fragments and labelled with codes. New codes were added to the existing codebook in a constant comparative process, in which codes generated by each of the three data coders were discussed during a series of meetings, to reach consensus and resolve discrepancies. Once all the transcripts were saturated of all codes, inter-relationships between codes were identified to ascertain over-arching themes. Finally, contrasts and comparisons between groups were identified.

## Results

### Study participants

Twenty-five FND patients were recruited. Three were subsequently excluded due to medical comorbidity that could interfere with the study results. Twenty caregivers were recruited. Two were then excluded as FND turned out to not be the main problem leading to disability in the patient, and one because the person was known to the research team. All 21 recruited HCPs were included.

Descriptives of study participants are displayed in Table 1, with details in the Supplementary Materials. The majority of FND patients had mixed symptoms, about a quarter had only seizures, one patient had only movement and one only sensory symptoms. Mean duration of FND was five years. Of the caregivers, 72% were partners and 28% were parents of the FND patient. The HCP group consisted of neurologists, neuropsychiatrists, psychologists, physiotherapists, nurses and a speech and language therapist. Mean duration of experience working with FND patients was 10 years.

**Table 1:** Characteristics of study participants

	FND patients (*n* = 22)		Caregivers (*n* = 18)		HCPs (*n* = 21)	
	Mean (SD)	*n* (%)	Mean (SD)	*n* (%)	Mean (SD)	*n* (%)
Age (years)	42 (13)		50 (14)		42 (11)	
Gender ·Female ·Male		17 (77%) 5 (23%)		12 (67%) 6 (33%)		16 (76%) 5 (24%)
Marital status ·Married/cohabiting ·Single		16 (73%) 6 (27%)				
Employment status ·Employed ·Unemployed ·Student ·Medical leave/retired		9 (41%) 5 (23%) 1 (0.5%) 7 (32%)		12 (67%) 0 (0%) 0 (0%) 6 (33%)		
Symptom type ·Movement disorder ·Seizures/attacks ·Sensory ·Mixed		1 (5%) 5 (23%) 1 (5%) 15 (68%)				
Comorbidity* ·Psychiatric ·Physical ·None		9 (41%) 5 (23%) 11 (50%)				
Duration FND (months)	66 (56)					
Duration caregiver (months)			51 (40)			
Relationship ·Parent ·Partner/spouse				5 (28%) 13 (72%)		
Full-time/part-time caregiver Full-time Part-time				8 (44%) 10 (56%)		
Profession ·Neurologist ·Neuropsychiatrist ·Physiotherapist ·Psychol/Psychoth ·Occupational therapist ·Speech & Language Therapist ·Nurse						4 (19%) 4 (19%) 3 (14%) 4 (19%) 3 (14%) 1 (5%) 2 (10%)
Service type^a^ ·Specialist inpatient ·Specialist outpatient ·General inpatient ·General outpatient						12 (57%) 12 (57%) 5 (24%) 3 (14%)
Duration professional experience (months)					192 (133)	
Duration FND experience (months)					125 (118)	

HCP: health care professional; psychol/psychoth: psychologist, psychotherapist or CBT therapist

^a^Multiple answers were possible

### Treatment goals

#### Treatment goals

In total, 21 treatment goals were mentioned by the study participants, which could be categorised as physical, psychological, social, functional/participation, quality-of-life and healthcare (Table 2).

**Table 2:** Treatment goals and hopes for outcomes of FND treatment, described as the number and percentage of participants within of each group that mentioned this topic [N(%)] and the number of times the topic was mentioned in the group in total (Ntot). The top five treatment goals (i.e. mentioned by the largest percentage of each group) are printed in bold font

Treatment goal	N(%) patients (*n* = 22)	N(%) caregivers (*n* = 18)	N(%) HCPs (*n* = 21)	Ntot patients	Ntot caregivers	Ntot HCPs
Physical						
Complete and sustained resolution of all symptoms	**8 (36%)**	**8 (44%)**	6 (29%)	9	11	7
Reduction or improvement of functional neurological symptoms	**8 (36%)**	**8 (44%)**	**11 (52%)**	16	11	17
Reduction or improvement of commonly co-occuring symptoms	4 (18%)	4 (22%)	3 (14%)	4	4	4
Increased physical health/well-being	1 (5%)	0 (0%)	1 (8%)	1	0	1
Psychological						
Understanding and acceptance of diagnosis	6 (27%)	3 (17%)	**9 (43%)**	10	3	12
Understanding of underlying cause(s) of FND	0 (0%)	0 (0%)	5 (24%)	0	0	10
Increased mental health/well-being	**13 (59%)**	**6 (33%)**	**14 (67%)**	23	12	20
Social						
Improvements in close relationships	2 (9%)	3 (17%)	4 (19%)	2	3	6
Improved social functioning	3 (14%)	1 (6%)	2 (10%)	4	1	2
Functional/participation						
The ability to return to work/volunteer	**8 (36%)**	5 (28%)	2 (10%)	9	8	2
The ability to resume education	0 (0%)	1 (6%)	1 (8%)	0	1	1
The ability to resume hobbies	3 (14%)	1 (6%)	0 (0%)	3	1	0
Increased independence	2 (9%)	3 (17%)	**11 (52%)**	2	3	19
Improvement in impairment/disability	6 (27%)	1 (6%)	7 (33%)	8	1	11
Improved mobility	6 (27%)	5 (28%)	6 (29%)	7	5	7
Increased self-management/self-efficacy	**10 (45%)**	**6 (33%)**	**16 (76%)**	16	7	26
Return to baseline functioning	2 (9%)	5 (28%)	5 (24%)	2	5	7
Improved functioning (non-specified)	1 (5%)	1 (6%)	7 (33%)	1	1	7
Quality of life						
Improved quality of life	1 (5%)	**6 (33%)**	6 (29%)	1	7	8
Health care						
Prevention of unnecessary medical interventions	0 (0%)	0 (0%)	2 (10%)	0	0	2
Reduced health care costs	0 (0%)	0 (0%)	3 (14%)	0	0	4

Whilst an improvement in, or complete and sustained resolution of, functional neurological symptoms were in the top three treatment goals of all groups, this was mentioned as a treatment goal by a larger proportion of caregivers and HCPs than patients. Patients most frequently hoped that treatment would result in an increase in mental health/well-being. This was also amongst the treatment goals reported by the largest number of caregivers and HCPs. Several patients mentioned that they developed anxiety and feelings of depression due to FND, expressing a wish to learn how to cope with the uncertainties resulting from their disorder. Patient 01–11: “…*and I think the other thing I’ve missed is dealing with the anxiety and the uncertainty of it, […] that's the bit that I think I struggle with most, is that, what's the future going to…kind of hold…*”.

Another important treatment goal for patients was the ability to return to (volunteer) work. Patient 01–03: *“I’ve got four and a half years before I retire and I'd like them to be productive years, because I like working in [work], I like helping people—I want to get back to work.”*

HCPs most frequently reported an increase of self-management or self-efficacy as a treatment goal. They mentioned that an important focus of their treatment was providing patients with a set of self-management tools and a relapse prevention plan. HCP 03–15: *“[…] and if they do have some kind of relapse, that we've provided them with the tools to kind of look back and use those tools to regain independence again […] without the need to come in for an inpatient admission again, so hopefully they can, they can move forwards at home.”* Increased self-management and self-efficacy were also amongst the most frequently reported treatment goals for patients and caregivers. Patient 01–17: “*I think “recovery” in quotes, for me, would be about having reached a stage in life where I’m managing my life so that I'm not triggering symptoms…”.*

Whilst an increase in independence was reported as one of the most important treatment goals of the HCPs, this was mentioned by a smaller percentage of patients and caregivers.

Several HCPs reported that a reduction of healthcare costs and the prevention of unnecessary medical interventions were important outcomes. They tried to achieve this by explaining the diagnosis adequately. This is in line with another treatment goal mentioned by many HCPs: an understanding and acceptance of the diagnosis.

#### Setting treatment goals

A large percentage of participants in all groups discussed the importance of setting realistic treatment goals instead of striving for the ideal outcome (64% of patient, 56% of caregivers and 52% of HCPs). Multiple patients and caregivers mentioned that they did not expect full recovery anymore, but aimed to achieve other treatment goals, like a reduction of functional neurological symptoms, or the ability to perform certain activities again. Patient 01–07: “*Well, I don't think I can say that I will never have this again, and I think if I, if I, if I put myself in that mindset that I will never, ever have any kind of FND symptoms again, then I think I'd be setting myself up to fail. I think it's more about being pain free and having that independence, that I would say I'm, I'm…recovered.*” HCPs often mentioned that expectation management of treatment outcomes was an important first step in treatment. HCP 03–13: “*So, you have some people say ‘I don’t think anything can possibly be done’…and you think well this is entirely treatable […] and maybe sometimes you say ‘Look, you have been like this for 10 years, maybe we have to be realistic about what we can really try to do, maybe try to take these aims down a bit.”*

Eleven per cent of caregivers and 9% of patients remarked that treatment goals should be patient-centred. This aligned with the HCPs’ approach: 38% of them spontaneously reported that they would record patient-centred treatment goals at the start of treatment, and would evaluate these over the course of treatment. HCP 03–13: “*I think what I've started to realize over the years is patients are actually quite good at telling you what they want, whereas I used to battle away thinking everyone was trying to get better*”.

### Outcome measurement of FND

#### Outcomes

Participants reported a total of 20 outcomes deemed relevant for clinical outcome assessment of FND. These outcomes were categorised into a physical, psychological, social, functional/participation, quality-of-life and healthcare outcome domain (Table 3). The outcomes mentioned by the largest percentage of patients were functional neurological symptoms, emotional well-being and the achievement of treatment goals. The largest percentage of caregivers and HCPs similarly mentioned functional neurological symptoms and emotional well-being, but caregivers also mentioned achievement of treatment goals as an important outcome, whilst HCPs also reported activities of daily living and quality-of-life frequently.

**Table 3:** Outcomes relevant for FND clinical assessment, described as the number and percentage of participants within each group that mentioned this topic [N(%)], as well as the number of times the topic was mentioned in this group [N(tot)]. The top three outcomes (i.e. mentioned by the largest percentage of each group) are printed in bold font

Outcomes	N(%) patients (*n* = 22)	N(%) caregivers (*n* = 18)	N(%) HCPs (*n* = 21)	N(tot) patients	N(tot) caregivers	N(tot) HCPs
Physical						
Functional neurological symptoms	**18 (82%)**	**7 (66%)**	**11 (52%)**	22	9	14
Triggers	3 (14%)	2 (11%)	0 (0%)	4	2	0
Co-occuring symptoms	3 (14%)	4 (22%)	3 (14%)	5	6	3
Physical well-being	2 (9%)	2 (11%)	2 (9%)	2	4	2
Psychological						
Emotional well-being	**10 (45%)**	**7 (66%)**	**9 (43%)**	13	14	13
Illness-related beliefs/insight	2 (9%)	1 (6%)	2 (9%)	6	1	3
Social						
Relationships	2 (9%)	1 (6%)	5 (24%)	2	1	5
Social functioning	3 (14%)	1 (6%)	4 (19%)	5	1	4
Functional and participation						
Activities of daily living	6 (27%)	**6 (33%)**	**9 (43%)**	9	8	16
Work	3 (14%)	2 (11%)	6 (29%)	4	2	9
School/education	0 (0%)	0 (0%)	1 (5%)	0	0	1
Travel/driving	2 (9%)	0 (0%)	0 (0%)	4	0	0
Mobility	2 (9%)	2 (11%)	2 (9%)	2	2	4
Finances	1 (5%)	2 (11%)	0 (0%)	1	2	0
Recreational activities	5 (23%)	8 (14%)	4 (19%)	5	14	6
Level of dependency	3 (14%)	1 (6%)	0 (0%)	3	1	0
Quality of life						
Quality of life	5 (23%)	4 (22%)	**8 (38%)**	7	5	9
Health care						
Achievement of treatment goals/self-perceived improvement	**7 (32%)**	**6 (33%)**	5 (24%)	9	6	8
Health economics	1 (5%)	0 (0%)	5 (24%)	1	0	5
Self-management	4 (18%)	0 (0%)	1 (5%)	4	0	1

#### Outcome assessment

None of the participants thought that outcome measurement should be purely clinician-rated or objective. Whilst the largest proportion of participants (91% of patients, 83% of caregivers and 86% of HCPs) thought that outcome measurement of FND should be rated by patients as well as the HCPs, all participants believed that the patient’s experience should be central. HCP 03–07 says: “*I mean, look, at the end of the day, an improvement doesn't matter if patients don't experience it as an improvement, right, so in a way, I think the subjective experience is the most important.*”

However, participants in all groups acknowledged that HCPs have an important role in outcome assessment as well, and that objective assessments have added value, as there can be a discrepancy between subjective and objective functioning. HCP 03–19: “*Occasionally we get patients who, who feel just as bad, even though we can see objectively on the ward that they're walking a bit better, they’re doing things that they don't recognize that they're doing.”*

Some participants mentioned that it was important to also include the caregiver’s views in the assessment. Caregiver 02–12: “*I can observe over a long period of time how it's going and how it's like in daily life.”*

#### Setting of assessment

When asked about the preferred setting of the outcome assessment, most patients reported that they would prefer to conduct the assessment together with another person. This could be either with a HCP or caregiver who would help them complete the OMIs (14%), in a dialogue with their HCP (27%), or in a meeting with their multidisciplinary treatment team (14%), as a periodic treatment evaluation. From the HCP group, 14% felt the FND patients should fill out the OMIs alone for practical reasons to obtain an independent rating from the patient, whilst 24% thought the patient and HCP(s) should fill these out together. Over two-thirds of caregivers felt they should be included in the assessment.

#### Design of outcome measurement instrument

Several participants mentioned that an OMI should be adjusted to the patient’s disabilities. In that regard, participants commented on the advantages of digital outcome assessment. Patient 01–08: “*One of the problems that I've got is that I can't write anymore with a pen […]. So the ability to fill it out on an iPad or a smartphone, something like that, would be far easier for me than filling out something by longhand.”* Moreover, between a third and half of participants in each group mentioned the importance of keeping self-reported OMIs, i.e. questionnaires, short and simple. Multiple patients mentioned they preferred questions with different response options, i.e. tick-box questions supplemented with open-text response options. Caregiver 02–03: *“I think…having an area where, let's say paralysis, and then you score 0 to 10, and maybe have a box next to it, […] Just to kind of, because, I don't know, if I was paralyzed on the lefty side, I might be left-handed and therefore I wouldn't be able to write, [..] So, just to kind of clarify, I suppose.*”

A couple of participants also mentioned that outcome measurement in FND should be personalised, i.e. in line with a patient’s unique symptoms or treatment goals. Caregiver 02–20: “*I think it’s the kind of illness where you absolutely have to have an individualized approach, because of the way it impacts on people. So even in [hospital], there was a girl in there who seemed to have similar symptoms to [daughter], but they were impacting on her differently, and yet they were around the same age and stuff like that. So I think because it does seem to impact on people differently, we cannot have a one size fits all.*”

## Discussion

This is the first qualitative study to capture the views of FND patients, their caregivers and HCPs on outcome measurement in FND. We conducted in-depth qualitative interviews to explore the views of all key stakeholders on treatment goals, relevant outcomes, and how best to measure these outcomes.

Study participants reported a total of 21 treatment goals during the interviews. Treatment goals mentioned by the largest number of participants were an improvement in or complete and sustained resolution of functional neurological symptoms, an increase in mental health/well-being, self-management or self-efficacy, independence and quality-of-life, and the ability to return to work or volunteering. Interestingly, whilst the assessment of functional neurological symptoms was deemed important by the majority of patients, improvement or resolution of functional neurological symptoms was not the treatment goal mentioned most frequently by this group; patients most frequently hoped that treatment would result in an increase in mental health/well-being. Clinical trials have tended to focus on symptom reduction. For example, a recent large trial of the efficacy of cognitive behavioural therapy (CBT) focussed on functional neurological symptoms, with monthly seizure frequency as the primary outcome [10]. Whilst no significant differences in seizure frequency were observed between the CBT and control group, patients in the CBT group reported significantly greater improvement of self-perceived health as well as distress, seizure bothersomeness, and functioning. This suggests that the ability to cope with the seizures might be more relevant to self-perceived health than the frequency of symptoms, which aligns with the results from our study. Other qualitative studies on the experiences of FND patients demonstrate the substantial negative impact of FND on mental well-being, including distress and frustration related to coping with symptoms [11, 12]. Psychological support, focussed on dealing with the impact of FND, might therefore be important for the well-being of FND patients, particularly those with chronic or severe symptoms.

When asked which outcomes are relevant for FND outcome assessment, the study participants reported 20 outcomes that spanned several domains: physical, psychological, social, functional/participation (including work/study), quality-of-life and healthcare. The specific outcomes mentioned by the largest percentages of participants were functional neurological symptoms, emotional well-being, the ability to perform activities of daily living and recreational activities, the achievement of treatment goals, and quality-of-life. These correspond largely to those in existing outcome measure systems [13, 14], suggesting that relevant outcomes in FND are quite similar to those for other medical and mental health disorders. However, they also align with the FND-specific interim core outcome set that was formulated by the by the FND-COM group [5], recommending assessment of functional neurological symptoms, other physical symptoms, psychological symptoms, life impact, health economics and adverse events in FND clinical trials.

As mentioned by some of the study participants, FND outcome measurement can be complicated due to discrepancies between FND patients’ self-reported and clinician-rated symptom severity or function [6]. One of the diagnostic features of FND is inconsistency, referring to the observation that attention tends to exacerbate symptoms whilst distraction can reduce them, which can result in an overestimation of FND severity during assessments. Whilst there is a traditional preference for objective OMIs in clinical outcome assessment, more recently developed core outcome sets for neurological and functional disorders include prominent patient-reported OMIs [15, 16], and FND experts agree that patient-reported OMIs should be prioritised over objective OMIs [6]. In this qualitative study, too, all participants agreed that the patient’s subjective experience should be central in clinical assessment and evaluation of treatment effects. However, participants in all groups also acknowledged the value of clinician-rated or objective OMIs.

The majority of patients in this study preferred to conduct the outcome assessment together with one or multiple HCP(s), so they could get assistance around and/or discuss the outcomes. However, a number of participants also believed outcome assessment could be performed digitally. In other medical disorders, digital OMIs are also preferred by patients over paper-based methods [17]. Each group mentioned the importance of keeping self-reported OMIs short and simple. The Patient-Reported Outcomes Measurement Information System (PROMIS), the US National Institute of Health’s databank of OMIs for self-reported health-related constructs, uses computer-adaptive tests (CAT) [18]. CAT is a form of computer-based testing that adapts the number and type of questions to the respondent’s previous answers, reducing respondent burden [18]. CAT might be an useful option for FND outcome assessment in the future.

This is the first study on the views of patients, their caregivers and HCPs on outcome measurement in FND. The strengths are the thorough methodology used, as well as the large sample sizes for a qualitative study of three different key ‘stakeholder’ groups, i.e. patients, their caregivers and a full and representative range of HCPs. A possible limitation of this study is the relatively long duration of FND symptoms of participants, with 91% of FND patients having symptoms for more than a year. One can imagine that patients with a short illness duration still hope for a complete resolution of symptoms, whilst chronic FND patients may focus more on other treatment outcomes, like mental well-being and quality-of-life.

This study is an important addition to previous publications describing FND expert opinion on FND clinical outcome assessment [5, 6]. The results highlight the need for patient-centred OMIs for FND, that also allow input from HCPs, which focus on monitoring change in FND symptoms, mental health/well-being, the ability to perform activities of daily living and quality-of-life. Our findings will facilitate the integration of patients’, caregivers’ and HCPs’ views in the future development of FND-specific OMIs or a core outcome set for this disorder.

## Supplementary Information

Below is the link to the electronic supplementary material.

Supplementary file1 (DOCX 31 KB)

## Data Availability

Not applicable.
