# Supplementary material for: Outcome measurement in functional neurological disorder: A qualitative study on the views of patients, caregivers and healthcare professionals
Source: J Neurol. 2025 Feb 11;272(3):189. doi: 10.1007/s00415-025-12912-9 (PMC11814038; doi:10.1007/s00415-025-12912-9)
Supplement: Supplementary file 1 — Supplementary file1 (DOCX 31 KB) [file 415_2025_12912_MOESM1_ESM.docx]

***Supplement 1***

Title: Outcome measurement in functional neurological disorder: a qualitative study on the views of patients, caregivers and healthcare professionals

Journal: Journal of Neurology

Authors: Sonja Rutten, Abigail Bradley-Westguard, Timothy R. Nicholson, Susannah Pick

Corresponding author: Susannah Pick, King’s College London (Institute of Psychiatry, Psychology & Neuroscience, Department of Psychological Medicine), London, United Kingdom. E-mail: susannah.pick@kcl.ac.uk

**Table 1: Sociodemographic and clinical characteristics of FND patients**

| **Participant number** | **Age** | **Gender** | **Marital Status** | **Employment status** | **Comorbid Psych Dx** | **Comorbid somatic Dx** | **Duration FND (months)** | **Symptom type** | **Duration since Dx (months)** |
| --- | --- | --- | --- | --- | --- | --- | --- | --- | --- |
| **01-01** | [18-24] | F | Single | Unemployed | n.a. | n.a. | 8 | Mixed | 2 |
| **01-02** | [35-44] | M | Married/Cohab | Unemployed | n.a. | n.a. | 98 | Mixed | 7 |
| **01-03** | [55-64] | F | Single | Unemployed | ED | n.a. | 14 | Mixed | 5 |
| **01-04** | [18-24] | F | Married/Cohab | Employed | n.a. | n.a. | 28 | Mixed | 2 |
| **01-05** | [35-44] | M | Married/Cohab | Unemployed | n.a. | n.a. | 26 | Sensory | 17 |
| **01-06** | [45-54] | F | Single | Employed | n.a. | n.a. | 149 | F/DS | 10 |
| **01-07** | [45-54] | F | Married/Cohab | Medical leave/retired | n.a. | n.a. | 46 | Mixed | 39 |
| **01-08** | [55-64] | M | Married/Cohab | Medical leave/retired | n.a. | n.a. | 168 | Mixed | 72 |
| **01-11** | [25-34] | F | Married/Cohab | Employed | AD | n.a. | 19 | Mixed | 19 |
| **01-13** | [35-44] | F | Married/Cohab | Student | n.a. | n.a. | 36 | F/DS | unknown |
| **01-14** | [55-64] | F | Single | Medical leave/retired | AD | n.a. | 13 | FMD | 1 |
| **01-15** | [25-34] | F | Single | Employed | AD | n.a. | 28 | Mixed | 26 |
| **01-16** | [45-54] | F | Married/Cohab | Unemployed | n.a. | DM, IBS | 156 | Mixed | 36 |
| **01-17** | [35-44] | F | Married/Cohab | Employed | ED | Migraine | 180 | Mixed | 48 |
| **01-18** | [45-54] | M | Single | Medical leave/retired | n.a. | n.a. | 54 | Mixed | 14 |
| **01-19** | [45-54] | F | Married/Cohab | Medical leave/retired | AD | CFS, pulm | 37 | Mixed | 37 |
| **01-20** | [25-34] | F | Married/Cohab | Employed | AD | n.a. | 108 | F/DS | 72 |
| **01-21** | [45-54] | M | Married/Cohab | Medical leave/retired | n.a. | n.a. | 36 | Mixed | 12 |
| **01-22** | [45-54] | F | Married/Cohab | Unemployed | n.a. | CRPS | 36 | Mixed | 24 |
| **01-23** | [35-44] | F | Married/Cohab | Employed | n.a. | n.a. | 10 | F/DS | 5 |
| **01-24** | [55-65] | F | Married/Cohab | Medical leave/retired | AD, PTSD | Pulm, rheu | 72 | F/DS | unknown |
| **01-25** | [25-34] | F | Married/Cohab | Unemployed | AD, PTSD | n.a. | 120 | Mixed | 8 |

*AD = affective disorder, CFS = chronic fatigue syndrome, Cohab = cohabiting, CRPS = complex regional pain syndrome, DM = diabetes mellitus, Dx = diagnosis, ED = eating disorder, F = female, F/DS = functional/dissociative seizures; FND = functional neurological disorder, FMD = functional movement disorder, IBS = irritable bowel syndrome, M = male, pulm = pulmonary condition, PTSD = posttraumatic stress disorder, rheu = rheumatologic condition, F/DS = functional / dissociative seizures*

**Table 2: Sociodemographic characteristics of caregivers**

| **Participant number** | **Age** | **Gender** | **Employment status** | **Duration FND caregiver (months)** | **Relationship to person with FND** | **Fulltime or part-time caregiver** |
| --- | --- | --- | --- | --- | --- | --- |
| **02-01** | [45-54] | F | Employed | 8 | Parent | FT |
| **02-02** | [35-44] | F | Employed | 23 | Partner / spouse | FT |
| **02-03** | [35-44] | F | Employed | 48 | Partner / spouse | FT |
| **02-05** | [45-54] | F | Employed | 30 | Parent | PT |
| **02-06** | [55-64] | M | Medical leave/retired | 37 | Partner / spouse | PT |
| **02-07** | [55-64] | F | Employed | 96 | Partner / spouse | PT |
| **02-08** | [25-34] | M | Employed | 60 | Partner / spouse | PT |
| **02-09** | [55-64] | F | Employed | 36 | Partner / spouse | PT |
| **02-10** | [55-64] | M | Medical leave/retired | 36 | Parent | FT |
| **02-12** | [65-75] | M | Medical leave/retired | 72 | Partner / spouse | PT |
| **02-13** | [25-34] | F | Employed | 120 | Partner / spouse | PT |
| **02-14** | [55-64] | F | Medical leave/retired | 26 | Partner / spouse | FT |
| **02-15** | [25-34] | F | Employed | 9 | Partner / spouse | PT |
| **02-16** | [35-44] | F | Employed | 8 | Partner / spouse | PT |
| **02-17** | [55-64] | M | Medical leave/retired | 4 | Partner / spouse | FT |
| **02-18** | [45-54] | F | Employed | 132 | Parent | FT |
| **02-19** | [55-64] | M | Medical leave/retired | 96 | Partner / spouse | FT |
| **02-20** | [55-64] | F | Employed | 84 | Parent | PT |

*F = female, FND = functional neurological disorder, PT = part-time, FT = fulltime, M = male*

**Table 3: Sociodemographic and professional characteristics of healthcare professionals**

| **Participant number** | **Age** | **Gender** | **Profession** | **Service type** | **Duration profession experience (months)** | **Duration FND experience (months)** |
| --- | --- | --- | --- | --- | --- | --- |
| **03-01** | [25-34] | F | Physio | Specialist inpatient | 72 | 30 |
| **03-02** | [55-64] | M | Neuropsych | Specialist inpatient / Specialist outpatient | 420 | 384 |
| **03-03** | [25-34] | F | SALT | Specialist inpatient | 72 | 15 |
| **03-04** | [25-34] | F | OT | Specialist outpatient | 48 | 22 |
| **03-05** | [25-34] | F | Physio | Specialist outpatient | 60 | 25 |
| **03-06** | [45-54] | F | OT | Specialist inpatient | 216 | 120 |
| **03-07** | [35-44] | F | Neurologist | Specialist outpatient / General outpatient / General inpatient | 216 | 216 |
| **03-08** | [35-44] | F | Psychol/psychoth | Specialist outpatient | 108 | 72 |
| **03-09** | [25-34] | F | Psychol/psychoth | Specialist inpatient | 84 | 12 |
| **03-10** | [35-44] | M | Neurologist | Specialist outpatient / General outpatient / General inpatient | 84 | 84 |
| **03-11** | [35-44] | M | Physio | Specialist outpatient | 180 | 108 |
| **03-12** | [45-54] | F | Psychol/psychoth | General outpatient | 72 | 18 |
| **03-13** | [45-54] | M | Neuropsych | Specialist inpatient / Specialist outpatient | 288 | 264 |
| **03-14** | [25-34] | M | Neuropsych | Specialist inpatient / Specialist outpatient | 144 | 60 |
| **03-15** | [35-44] | F | OT | Specialist inpatient | 168 | 42 |
| **03-16** | [35-44] | F | Neurologist | Specialist inpatient / Specialist outpatient / General inpatient / General outpatient | 84 | 84 |
| **03-17** | [45-54] | F | Nurse | Specialist inpatient | 288 | 216 |
| **03-18** | [45-54] | F | Neurologist | Specialist inpatient / Specialist outpatient / General inpatient / General outpatient | 348 | 240 |
| **03-19** | [55-64] | F | Neuropsych | Specialist inpatient / Specialist outpatient / General inpatient / General outpatient | 432 | 156 |
| **03-20** | [55-64] | F | Nurse | Specialist inpatient | 468 | 396 |
| **03-21** | [55-64] | F | Psychol/psychoth | Specialist inpatient | 180 | 60 |

*F = female, M = male, OT = occupational therapist, physio = physiotherapist, psychol/psychoth = psychologist, psychotherapist or CBT therapist, SALT = speech and language therapist*
